# Dvla Status Audit Campbell Centre, Milton Keynes

**DOI:** 10.1192/bjo.2026.11682

**Published:** 2026-06-30

**Authors:** Akinwande Onafalujo, Noman Khan, Palwasha Rehman, Aarushi Kaushal

**Affiliations:** Central and North West NHS Foundation Trust, Milton Keynes, United Kingdom

## Abstract

**Aims:**

- To identify and highlight the current practice of doctors at Campbell Centre relating to the driving status of the patients
- To raise awareness of any deficiencies identified
- To serve as stimulus for a change process – possibly via a Quality Improvement Project.

**Methods:**

2 audits done retrospectively for the periods of 01 - 30 June 2025 and 01 - 30 November 2025.

Sample population drawn from both Hazel (male acute inpatient) and Willow (female acute inpatient) Wards in the Campbell Centre Milton Keynes.

Sample sizes: June 2025 - 62 patients (32 male, 30 female), November 2025 - 59 patients (33 male, 26 female)

Driving status of all patients admitted in those time periods was examined by checking Progress Notes on Electronic Medical Records (SystmOne) throughout that period.

Two categories of searches were conducted: 1. Whether patients were advised of the need to inform DVLA of their mental health status. 2. If it was confirmed from patients that the DVLA had been informed by them.

This data was then collated and presented in percentages of; DVLA Advised, and DVLA Informed. The results of the 1st Audit were presented on 15th October 2025 at the General MDT of the Campbell Centre with recommendations made.

The data for June 2025 and November 2025 were then compared to demonstrate improvement in another presentation done on 28th January 2026 with issues identified and further recommendations made.

**Results:**

June 2025:

- DVLA Advised: Willow Ward - 7% compliance, Hazel Ward - 0% compliance
- DVLA Informed: Willow Ward - 0% compliance, Hazel Ward - 0% compliance

November 2025:

- DVLA Advised: Willow Ward - 27% compliance, Hazel Ward - 21% compliance
- DVLA Informed: Willow Ward - 5% compliance, Hazel Ward - 0% compliance

**Conclusion:**

- There was some improvement in compliance after the 1st audit presentation - awareness has been created
- Improvement was below expected level (50%) however and shows that this is not yet embedded as regular practice.
- Current practice continues to fall short of expected standard however, and there is thus scope for improvement and need for further re-audits

